# Associations between the circumstances and severity of head impacts in men’s university ice hockey

**DOI:** 10.1038/s41598-023-43785-5

**Published:** 2023-10-13

**Authors:** Olivia M. G. Aguiar, Tim R. Chow, Helen Chong, Omid Vakili, Stephen N. Robinovitch

**Affiliations:** Department of Biomedical Physiology and Kinesiology, Simon Fraser University, Burnaby, BC Canada

**Keywords:** Brain injuries, Trauma, Public health

## Abstract

Improved evidence on the most common and severe types of head impacts in ice hockey can guide efforts to preserve brain health through improvements in protective gear, rink design, player training, and rules of play. In this observational cohort study of men’s university hockey, we compared video evidence on the circumstances of 234 head impacts to measures of head impact severity (peak linear accelerations and rotational velocities) from helmet-mounted sensors (GForceTracker). Videos were analyzed with a validated questionnaire, and paired with helmet sensor data. Shoulder-to-head impacts were more common than hand- or elbow-, but there were no differences in head impact severity between upper limb contact sites (p ≥ 0.2). Head-to-glass impacts were nearly four times more common, and just as severe as head-to-board impacts (p ≥ 0.4). Head impacts resulting in major penalties (versus no penalty), or visible signs of concussion (versus no signs), involved greater head rotational velocities (p = 0.038 and 0.049, respectively). Head impacts occurred most often to the side of the head, along the boards to players in their offensive zone without puck possession. Head impact severity did not differ between cases where the head was (versus was not) the primary site of contact (p ≥ 0.6). Furthermore, penalties were called in only 4% of cases where the head was the initial point of contact. Accordingly, rules that focus on primary targeting of the head, while important and in need of improved enforcement, offer a limited solution.

## Introduction

Ice hockey has one of the highest rates of concussion among team sports^1–3^. Most concussions occur from player-on-player contact^2–5^ that results in a direct blow to the head^6,7^. Furthermore, there is growing evidence that repeated sub-concussive impacts (which are much more common than concussions in hockey) are associated with structural changes to the brain, and acute and chronic symptoms including depression, executive dysfunction, and cognitive impairment^8–10^. Preserving brain health requires efforts to reduce the frequency and severity of head impacts in ice hockey. There is a lack of understanding on the most common and most severe types of head impacts in ice hockey. This is a barrier to the design of strategies for protecting the brain through rule changes, skills training, and improvements in protective equipment and environmental design.

Research to date in hockey has focused primarily on sensor-based measures of head kinematics during game play^11–15^. These sensors, which are typically secured to the helmet or integrated into mouthguard, measure the peak linear acceleration and rotational acceleration (or velocity) of the head, which are measures of head impact severity associated with brain injury risk^16,17^. In men’s university hockey, Wilcox et al. (2014a) reported that the 50th percentile in peak head linear acceleration ranged from 15 to 17 g, and for rotational acceleration from 1454 to 1733 rad/s^2^ ^15^. Based on impact location estimates from the sensor, Brainard et al. (2012) and Wilcox et al. (2014a) found that players sustained more impacts to the side (~ 30%), back (~ 30%) and front (~ 30%) of the head than the top (~ 10%)^11,15^. Wilcox et al. (2014a) also reported that impacts to the back of the head resulted in greater 95th percentile linear head accelerations than impacts to the front or side, and impacts to the side of the head resulted in greater rotational head accelerations than impacts to the front^15^. A limitation of these studies is that the occurrence of head impacts was not verified through video and/or direct observation. Lack of video verification led to overestimation of direct head impacts by up to 35% for helmet-mounted sensors, and 92% for mouthguard sensors^18,19^. Moreover, these studies provide limited insight on how head impacts occur, and how head impact severity depends on situational factors (e.g., puck possession, playing zone) and behavioural factors (e.g., visible signs of concussion, anticipation of collision)—which may inform rule development and enforcement, and improved detection of injuries (e.g., concussion spotters)^17,18,20^.

A small number of studies have combined video with helmet sensor data to examine both the circumstances and severity of head impacts. In male youth hockey (under 17), board checks and mid-ice collisions accounted for 68% of direct head impacts^21^. Open-ice collisions resulted in greater head linear and rotational accelerations than collisions along the perimeter^22^, and anticipated head impacts were nearly six times more common but just as severe as unanticipated head impacts^22^. Moreover, head accelerations were greater during collisions that were penalized^23^ or when the head was directly struck^21^, but only 15–17% of head impacts resulted in penalties^23^. In men’s university ice hockey, Wilcox et al. (2014b) found that contact with the ice resulted in higher linear accelerations than contact with another player^24^. Moreover, Wilcox et al. (2014b) reported that 50% of head impacts were due to contact with another player, and 37% were due to contact with the glass/boards^24^. However, neither Swenson et al. or Wilcox et al. (2014b) examined the specific body parts striking the head^21,24^. They also did not separate head impacts to the boards (bottom part of rink enclosure, typically constructed with high density polyethylene) versus glass (shielding that extends above the boards, typically constructed with tempered glass or acrylic), and did not examine the specific body parts striking the head. These issues are relevant to protective equipment and environment (rink) design.

Other studies have conducted video analysis of the circumstances of head impacts in ice hockey. In male youth hockey, Butterfield et al. (2023) and Post et al. (2021) found that the head was most often struck by an opposing player’s shoulder or elbow, or by the glass^25,26^. Regulation of body checking and the “zero tolerance for head contact” policy decreased the number of physical/head contacts by up to 30%, but the effect on head impact magnitude has not been reported^27–29^. No study to our knowledge has examined the specific objects impacting the head in men’s university hockey, and how the object affects head impact severity as measured by wearable sensors. Nor have studies compared the severity of penalized versus unpenalized head impact events in men’s university hockey. Improved insight on these issues may inform rule changes and enforcement. Furthermore, previous studies were limited by the use of a single camera that followed the puck, and the high likelihood of missing impact events occurring outside of the camera’s field of view. Using five cameras to capture the entire ice surface, we recently reported that the head was impacted nearly twice as often by the (gloved) hand than the shoulder/upper arm or elbow/forearm, and the head impacted the glass four times more often than the boards^30^. Furthermore, we found that impacts to the side of the head were four times more common than impacts to the back or front.

In this observational cohort study, we collected and analyzed helmet-mounted sensor measures and video footage of head impacts captured over three seasons of home games in men’s university hockey to address the following questions: (1) What are the most common circumstances (or scenarios) for head impacts in ice hockey? (2) How do the scenarios differ in terms of peak head linear accelerations and rotational velocities? In addition to characterizing the circumstances of head impacts with previously examined factors (e.g., location on the ice, anticipation, and penalties), we include previously unexplored characteristics of head impacts including the specific object that impacts the head, puck possession, and visible signs of concussion.

## Methods

### Participants

Forty-six players (33 forwards and 13 defensemen) of the Simon Fraser University (SFU) Men’s Ice Hockey team (British Columbia Intercollegiate Hockey League) participated in the study. Measures were acquired over three consecutive seasons from 2016 to 2019. Written informed consent was obtained from each player (including permission to publish the information/images in an online open access publication), the study was approved the Research Ethics Board of SFU (approval number 20140294), and participation in the research was performed in accordance with the Declaration of Helsinki.

### Helmet sensor data

**Figure 1:**
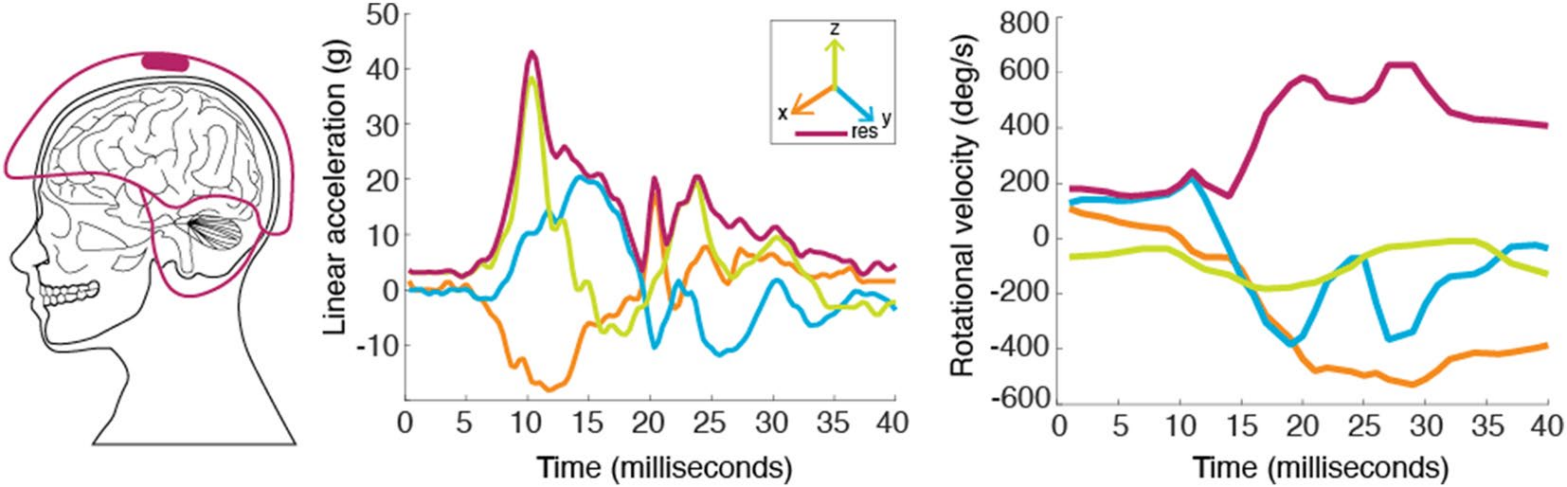
GForceTracker™ sensor placement in the helmet (left) and raw traces for linear acceleration (centre) and rotational velocity (right).

Participants were instrumented with helmet-mounted GForceTracker™ sensors (GFT; version 3.s.19; Artaflex, Markham, Canada) to measure head linear accelerations and rotational velocities during impact events. The GFT has been previously used in lacrosse^18,31^ and football studies^32^, and contains a triaxial accelerometer (measurement range of ± 200 g) and a triaxial gyroscope (measurement range of 2000°/s). The GFT sensors recorded, time stamped, and stored data in 40 ms segments (8 ms pre-trigger, 32 ms post-trigger), when any axis of the linear acceleration exceeded a user-defined threshold—which was set to 10 g for this study (recording threshold)^33^. Linear accelerations were sampled at 3000 Hz and rotational velocities were sampled at 800 Hz. Linear and rotational data were low pass filtered on-board with 300 Hz and 100 Hz anti-aliasing filters, respectively, and corrected to the centre of gravity of the head through built-in, proprietary sensor algorithms. Rotational velocities were converted from °/s to rad/s for comparison to other literature (Fig. 1).

Players wore the same helmet model (CCM Vector 08) to normalize the effect of helmet type and sensor location on the recorded sensor measures. Each player was assigned a specific GFT sensor, calibrated for their own helmet. The sensor was secured to the top right inside of the helmet’s shell with double-sided foam tape of 1.1 mm thickness (VHB, 3M, London, Canada). The sensor was turned on approximately 30 min before the start of the game and the real world time was noted. Time-stamped impact data were stored on-board and downloaded at the end of each game via gManager 1.8 software.

Recent studies have scrutinized the reliability and validity of helmet-mounted systems to accurately capture head kinematics during impact events^34–36^. Similar to the Head Impact Telemetry System (HITS)^37^, the accuracy of the head acceleration measured by the GFT depends on characteristics of the helmet and the direction of the impact^38–40^. However, when compared to HITS, a key advantage of the GFT is its direct and more accurate measurement of rotational velocity^38,41^, which is a key determinant of risk for brain injury^42^.

### Video footage of head impacts

**Figure 2:**
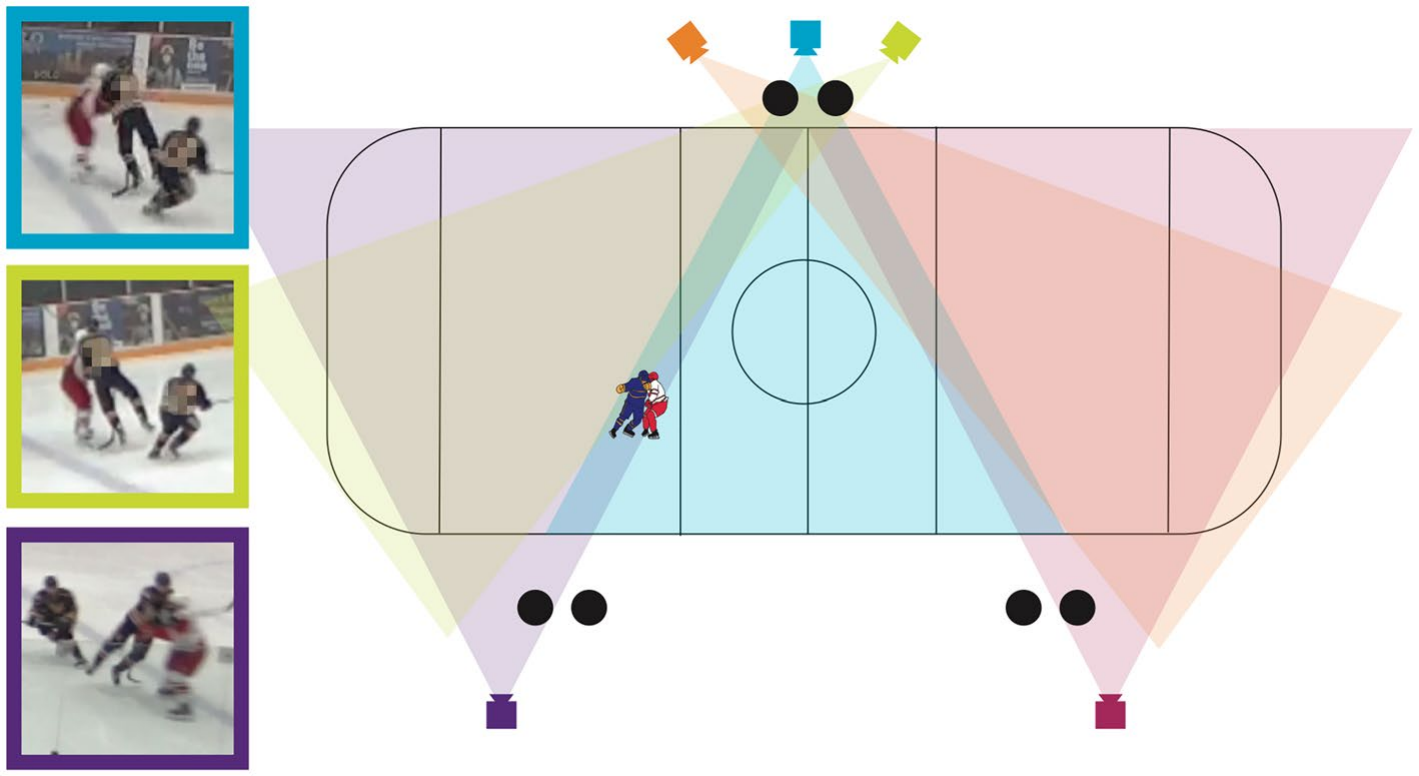
Five camera set-up with screenshots of a captured head impact event.

Five video camcorders (2 Sony HDR-CX330 and 3 Sony HDR-CX405BKIT), recording at 60 fps and 1920 × 1080 pixel resolution, were stationed around the ice surface to record the play from 25 home games (Fig. 2). This number of games has been shown in previous research to provide enough data to detect differences between categories of less than 10% in impact severity^33^. Six trained research assistants, including two at each of the three locations of the cameras, turned on the cameras at the start of the game (indicated by the referees’ audible whistle). A member of the research team then flashed a laptop in front of each camera, showing the real-world time and date. This allowed us to time-synchronize the video and sensor data up to the closest second. During the game, each of the six research assistants watched the game from different angles around the rink, and recorded (in written game notes) the time, location, and jersey number for every head impacts they observed. A head impact was defined as visible contact by any external object or player applied directly to the struck player’s head.

### Synchronization of sensor and video data

We used the research assistants’ game notes (including the noted time of the impact) to search the video footage and confirm the occurrence of head impacts. We then identified and only included cases in our analysis where the GFT sensor recorded data that were time-stamped to within ± 10 s of the observed onset of head contact in the video. The ± 10 s window accounted for resolution and drift in the timestamps recorded by the video cameras and the GForcetracker sensors. In the event that more than one impact head impact was observed on video within the ± 10 s window, the event having the closest timestamp to sensor data was selected. For cases meeting the ± 10 s criteria, we extracted the peak head linear accelerations and rotational velocities from the raw GFT impact data. We included head-impact events that resulted in a peak linear acceleration ≥ 10 g (inclusion threshold). If no sensor data were present or timestamps differed by more than 10 s, the head impact was excluded from our analysis. Video footage of verified head impacts were clipped and edited in Adobe Premier Pro CS4 (San Jose, USA) for further analysis.

### Head impact video analysis

Each head impact video was analyzed independently by three trained raters using a reliable and validated questionnaire^43^. We previously describe the nature and inter-rater reliability of the questionnaire and procedures for training raters^30^. For the eight questionnaire items included in this study, the total percent agreement (TPA) and free marginal kappa^44^ ranged between 67 and 97% and 0.31–0.93, respectively. At least 2 of the 3 raters had to reach consensus to obtain the final answer for a questionnaire item. If no consensus was reached, a fourth rater selected the best perceived response. All clipped videos were analyzed using Quicktime (up to v10.4; Apple, Cupertino, USA) or VLC Media Players (up to v3.0.8; VideoLAN, Paris, France).

The questionnaire probed observable situational factors before, during and after impact to the head. We classified the following factors before the collision (Table 1): the playing zone, location on the ice where the head impact occurred, whether the player receiving the head impact was in possession of the puck and whether the player was looking in the direction of the collision. For location on the ice, the rink was divided into “perimeter” (directly adjacent or within approximately one meter from the side boards, corners, end boards), “open ice” (interior portion of ice not accounted for by perimeter), and “near the net” (circumference surrounding the net— including the crease area which is approximately 2.4 m in diameter). “No puck possession” included cases where the player just released the puck, was attempting to gain puck possession or had no possession of the puck.

**Table 1:** Estimated means and confidence intervals (CI) for peak linear accelerations and rotational velocities with respect to situational factors preceding head impact (playing zone, location on ice, puck possession, and direction of gaze), within ± 10 s of the reference time.

	Frequency	Linear acceleration (g)		Rotational velocity (rad/s)	
	Count (% of head impacts captured)	Mean (95% CI)	p value	Mean (95% CI)	p value
Playing zone*	n = 234	n = 234	0.468	n = 234	0.096
Offensive zone	128 (54.7%)	38.2 (25.3–57.7)		14.4 (13.9–14.9)	
Defensive zone	76 (32.5%)	31.6 (25.7–38.8)		13.6 (12.1–15.3)	
Neutral zone	30 (12.8%)	36.8 (26.5–51.2)		17.5 (14.5–21.2)	
Location on ice	n = 234	n = 234	0.385	n = 234	0.273
Perimeter	181 (77.4%)	34.6 (31.8–37.6)		14.4 (13.6–15.2)	
Open ice	41 (17.5%)	32.6 (26.5–40.2)		14.1 (12.3–16.2)	
Near the net (crease)	12 (5.1%)	47.6 (30.1–75.4)		18.8 (13.8–25.6)	
Puck possession*	n = 234	n = 234	0.987	n = 234	0.580
Clear possession	30 (12.8%)	35.2 (26.8–46.1)		14.0 (11.7–16.8)	
No possession**	204 (87.2%)	35.1 (33.1–37.2)		14.9 (14.3–15.5)	
Looking in direction of collision*	n = 234	n = 234	0.706	n = 234	0.919
Yes	141 (60.3%)	34.5 (31.7–37.6)		14.7 (13.9–15.6)	
No	93 (39.7%)	36.0 (31.2–41.5)		14.6 (13.3–16.1)	

*Relative to the player receiving the head impact. **No possession includes the player attempting to gain possession, just releasing the puck (e.g., shot or pass), or no clear possession of the puck.

We also classified the following factors during the head impact event (Table 2): the specific body part or object contacting the head, the aspect of the head that was struck, and whether the head was the initial site of contact. The object striking the head was classified as “hand,” “elbow/forearm,” “shoulder/upper arm,” “glass,” “board,” “ice,” “puck,” “net,” “head,” “torso,” or “lower limb.”

**Table 2:** Estimated means and confidence intervals (CI) for peak linear accelerations and rotational velocities with respect to situational factors at the instant of head impact (objects striking the head, location of impact on the head, and whether the head was the initial point of contact), within ± 10 s of the reference time.

	Frequency	Linear acceleration (g)		Rotational velocity (rad/s)	
	Count (% of head impacts captured)	Mean (95% CI)	p value	Mean (95% CI)	p value
Object impacting head	n = 234	n = 234	0.636	n = 234	0.758
Board/glass	85 (36.3%)	36.4 (31.0–42.7)		14.9 (12.8–20.2)	
Elbow/forearm/hand	61 (26.0%)	31.9 (26.3–38.7)		13.7 (11.7–16.1)	
Shoulder/upper arm	43 (18.4%)	35.0 (22.9–43.9)		16.1 (12.8–20.2)	
Head	19 (8.1%)	44.3 (30.3–64.6)		17.1 (13.1–22.3)	
Other*	12 (5.1%)	32.2 (20.2–51.6)		14.1 (9.9–19.9)	
Stick	11 (4.7%)	36.5 (23.3–57.0)		14.6 (8.8–24.0)	
Ice	3 (1.3%)	17.9 (6.8–47.4)		13.8 (7.3–26.2)	
Impact to environment object versus another player	n = 222	n = 222	0.922	n = 222	0.821
Body part	134 (60.4%)	35.5 (32.7–38.7)		14.5 (13.8–15.5)	
Environmental object**	88 (39.6%)	35.9 (30.7–42.0)		14.3 (12.9–15.8)	
Upper limb contact site***	n = 104	n = 104	0.860	n = 104	0.254
Hand	41 (39.4%)	34.0 (25.3–45.7)		13.5 (11.3–16.1)	
Shoulder/upper arm	43 (41.3%)	37.0 (29.2–46.6)		15.9 (13.9–18.3)	
Elbow/forearm	20 (19.2%)	31.5 (20.2–49.3)		12.1 (9.2–15.7)	
Glass versus boards/impacting head	n = 85	n = 85	0.742	n = 85	0.477
Glass	67 (78.8%)	37.4 (30.5–46.1)		14.7 (13.0–16.8)	
Board	18 (21.2%)	35.1 (24.4–50.5)		13.5 (10.8–16.8)	
Location of impact on head	n = 234	n = 234	0.171	n = 234	0.680
Side	146 (62.4%)	36.5 (22.5–39.8)		15.2 (14.3–16.1)	
Front	49 (20.9%)	28.7 (23.5–34.9)		13.8 (12.0–15.8)	
Back	28 (12.0%)	35.6 (26.6–47.5)		13.9 (11.3–17.0)	
Top (crown)	11 (4.7%)	48.2 (30.0–77.4)		15.7 (11.3–21.9)	
Head initial point of contact^†^	n = 223	n = 223	0.939	n = 223	0.812
No	169 (75.8%)	36.1 (31.0–42.0)		14.5 (13.6–15.4)	
Yes	54 (24.2%)	36.4 (28.3–46.9)		14.8 (13.2–16.6)	

*Where “other” consists of the “net,” “torso,” or “lower limb.” There were no cases of “puck.”; **Where “environment” consists of the “boards,” “glass,” and “ice.” Excludes n = 12 where n = 11 for stick and n = 1 for net; ***Upper limb contact site of player delivering the hit; ^†^Only includes cases involving another player (opponent or teammate).

Finally, we classified the outcome of the head impact (Table 3) based on: whether the player who received the head impact exhibited one or more visible signs of concussion, and whether the referee penalized the head impact event. We classified visible signs of concussion based on definitions provided by Echemendia et al. (2018) (“slow to get up,” “clutching of head,” “motor incoordination,” “loss of consciousness,” “blank or vacant look,” “disorientation,” “visible facial injury in combination with any sign.”)^45^. We also examined the video to determine whether referees signaled for a penalty after the resulting impact event. We matched box score data from the league’s website to volunteer game notes (jersey numbers, clock time, rink location) to identify the infraction type and duration of each penalty. A “minor infraction” was defined as two minutes in the penalty box whereas a “major infraction” was defined as more than two minutes. Player body mass and height for SFU and the opposing team were extracted from the league’s website. This study did not include monitoring of diagnosed concussions.

**Table 3:** Estimated means and confidence intervals (CI) for peak linear accelerations and rotational velocities with respect to situational factors proceeding head impact (visible signs of concussion, penalty), within ± 10 s of the reference time.

	Frequency	Linear acceleration (g)		Rotational velocity (rad/s)	
	Count (% of head impacts captured)	Mean (95% CI)	p value	Mean (95% CI)	p value
Presence of visible sign(s) of concussion	n = 234	n = 234	0.678	n = 234	0.049
No	209 (89.3%)	34.7 (32.4–37.2)		14.0 (13.4–14.7)	
Yes	25 (10.7%)	37.3 (28.2–49.2)		17.6 (14.6–21.2)	
Penalization of head impact^†^	n = 223	n = 223	0.821	n = 223	0.152
No	204 (92.7%)	36.0 (30.8–42.2)		14.2 (13.7–14.8)	
Yes	16 (7.3%)	37.8 (24.7–57.9)		17.6 (13.6–22.7)	
Penalty type*^,†^	n = 223	n = 223	0.838	n = 223	0.049
No infraction	207 (92.8%)	36.0 (30.8–42.2)		14.2 (13.7–14.8)	
Minor infraction	12 (5.4%)	35.1 (21.7–59.7)		13.9 (10.2–18.9)	
Major infraction	4 (1.8%)	46.1 (19.9–106.6)		28.3 (16.8–47.6)	

^†^Only includes cases involving another player (opponent or teammate); *Where “minor infraction” was defined as less than two minutes in the penalty box and “major infraction” was defined as more than 2 mins.

### Statistical analysis

We report descriptive statistics (counts, percentages) along with back-transformed means and 95% confidence intervals (CI) for each response category. We used Generalized Linear Mixed Models to examine whether the severity of head impacts (as measured by peak linear acceleration and peak rotational velocity) differed among situational (explanatory) variables. We fit the model for a gamma error distribution with a log-link function, to correct for positive skewness of the sensor data. Participant code was treated as a random effect, to account for repeated head impacts by a given individual. Tukey post-hoc tests were used to examine pairwise comparisons when significant effects were observed between the response categories. The significance level was set to alpha = 0.05. Statistical analyses were performed using the Generalized Linear Mixed Model Add-in in JMP v16.0 for Macintosh (SAS Institute Inc., Cary, USA)^46^.

## Results

### Overview of observed head impacts

Over the 25 home games, we captured and verified 234 head impact events (video footage paired with helmet-sensor data within ± 10 s of the reference (laptop) time). Of the 234 impacts, 222 impacts (95%) had timestamps within ± 5 s, and 162 impacts (69%) were within ± 2 s. Head impacts were experienced by 30 unique, instrumented players, including 9 defensemen (41 events), and 21 forwards (193 events). The average number of recorded head impacts per player per game was 0.31 (range = 0.04–1.84). The mean body mass and height of player receiving the head impact were 80.6 kg (SD = 8.6, range = 68.0–96.1) and 178.9 cm (SD = 7.5, range = 167.6–198.1). The mean body mass and height of the player delivering the head impact were 85.8 kg (SD = 7.7, range = 72.7–111.4) and 183.8 cm (SD = 5.6, range = 165.1–198.1). There were no differences between forwards and defensemen in both measures of head impact severity (p > 0.63), and in the number of head impacts per player (p = 0.19).

The distributions of peak head linear accelerations and rotational velocities were positively skewed, due to the 10 g recording threshold for the GFT sensor, as observed in other studies^15,22^. The median peak linear acceleration and rotational velocity were 24.1 g (25th–75th percentile = 16.4–45.8 g) and 13.2 rad/s (25th–75th percentile = 9.1–18.3 rad/s), respectively. The mean peak linear acceleration and rotational velocity data were 36.1 g (SD = 29.4) and 14.6 rad/s (SD = 8.2), respectively.

### Situational factors preceding impact to the head

#### Playing zone and location on the ice

In total, 55% (n = 128/234) of head impacts occurred in the offensive zone, 32% occurred in the defensive zone and 13% occurred in the neutral zone (Table 1). 77% of head impacts were observed along the “perimeter” of the rink (n = 181/234). “Open ice” and “near the net” accounted for 18% and 5% of head impacts, respectively. There were no differences in head impact severity between playing zones or locations on the ice.

#### Puck possession and direction of gaze

Players without puck possession experienced a 6.8-fold greater number of head impacts (n = 204/234) than those with possession (n = 30/234; Table 1). There were no differences in the impact severity for the player with (versus without) puck possession. In 60% of cases, the player was looking in the direction of the impeding collision (n = 141/234). There were no differences in the head impact severity for the player looking (versus not looking) in the direction of the impending collision.

### Situational factors observed at the instant of head impact

#### Objects associated with head impact

**Figure 3:**
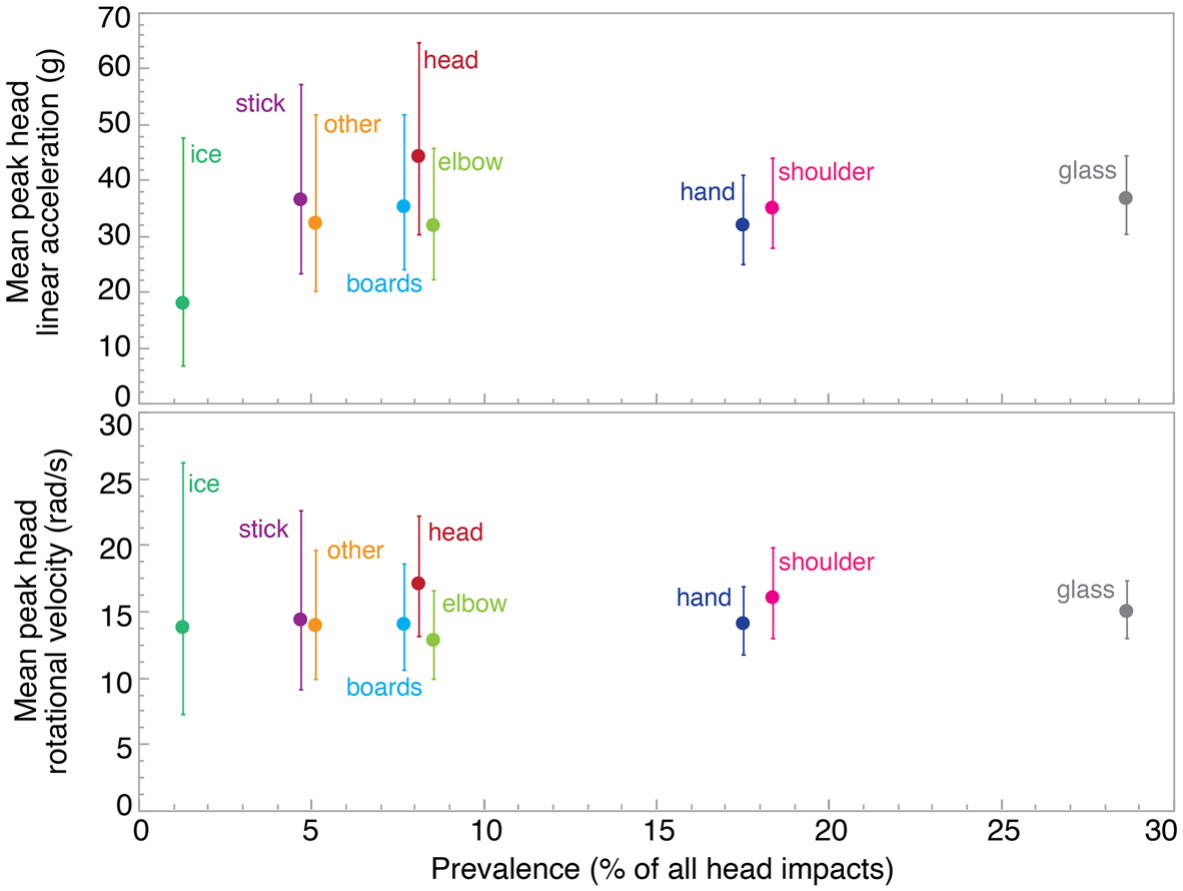
Mean peak head linear accelerations and rotational velocities as a function of the prevalence (percent of all head impacts) for each impacting object. The bars represent 95% confidence intervals. “Other” consists of the “net,” “torso,” and “lower limb.” There were no differences between impacting objects for head linear accelerations (p = 0.758) or rotational velocities (p = 0.636).

60% of head impacts were caused by contact with another players’ body part, whereas 40% were caused by contact with the environment—defined as glass, boards, and ice (Table 2, Fig. 3). There were no differences between impacting objects for head impact severity (p = 0.636 for rotational velocity, p = 0.758 for linear acceleration). “Shoulder/upper arm” and “hand” impacts to the head were over twice as common as “elbow/forearm.” Head impacts to the “glass” were 3.7 times more common than head impacts to “board.” There were no differences in head impact severity between upper limb contact sites, or between “glass” and “board.”

#### Impact location on head and head as the initial point of contact

In 62% of cases, players experienced an impact to the side of the head (Table 2). 21% of cases occurred to the front of the head, 12% to the back, and 5% to the top. There were no differences in impact severity between impact locations on the head. The head was the initial point of contact in 54 of 233 (24%) of cases involving contact with another player. There were no differences in head impact severity for cases where the head was not (versus was) the initial site of contact.

### Situational factors observed after head impact

#### Visible signs of concussion

We observed visible signs of concussion in 25 of 234 (11%) of cases (Table 3). “Slow to get up” was observed 21 times and “clutching of head” was observed 5 times. “Loss of consciousness,” “motor incoordination,” “blank or vacant look,” and “visible facial injury” were not observed. Of these cases, “board/glass” was the most common impacting object (n = 10/25), followed by the “shoulder/upper arm” (n = 4), “elbow/forearm/hand” (n = 4), and “stick” (n = 3). The mean peak rotational velocity (p = 0.049) was 1.3-fold greater when the player was (versus was not) visibly affected by the head impact, but there was no difference in the mean peak linear acceleration (p = 0.678).

#### Infraction

Penalties were called in 8% of the 223 head impacts involving a collision with another player (Table 3). 75% of these cases resulted in minor penalties. Only 4% of cases where the head was the initial point of contact were penalized. The mean peak rotational velocity was 2.0-fold higher for major infractions (penalties more than two minutes) than no infractions (p = 0.038), but there was no difference in rotational velocity between cases with minor infractions versus no infractions (p = 0.988) or major infractions (p = 0.074). There was no difference in mean peak linear acceleration for cases with versus without penalization of the head impact (p = 0.838).

## Discussion

To our knowledge, our study is the first to combine helmet-sensor measures with video footage to classify and examine how head impact severity depended on the circumstances of head impacts in men’s university ice hockey. We collected 234 head impact events and examined observable situational factors before, during and after the collision.

We found that the most severe head impacts tended to result in penalties. Player-on-player collisions resulting in “major infractions” (penalties of longer than two minutes in the box) generated 2.0-fold higher head rotational velocities than cases involving “no infraction.” Similarly, Mihalik et al. (2010b) found that, in male youth hockey, penalized impacts resulted in higher linear accelerations^23^. At the same time, we found multiple lines of evidence to support the notion that rules that focus on primary targeting of the head (e.g., Head Contact Rule 7.6 by Hockey Canada^47^), while important and in need of improved enforcement, offer a limited solution. First, direct targeting of the head rarely resulted in a penalty, indicating the challenge of reinforcing the rule. Only 4% of events where the head was the initial site of contact were penalized (n = 2 of 54). Second, when compare to primary head impacts, secondary impact to the head was far more common. Of the 234 head impacts we examined, the head was the initial site of contact in only 24% of cases. Finally, the severity of impacts did not depend on whether the head was the initial site of contact. Clearly, strategies are required to reduce the frequency and severity of both primary and secondary contacts to the head.

We found that players who exhibited visible signs of concussion (versus no signs) experienced impacts that produced 1.3-fold greater peak head rotational velocities. This finding casts doubt on the controversial question of whether players tend to purposefully exhibit visible signs of concussion for competitive advantage. The most common signs were “slow to get up” and “clutching of head.” Echemendia et al. (2018) and Bruce et al. (2018) examined the use of visible signs to predict subsequent concussion diagnosis in professional ice hockey^20,45^. They found that, despite being observed frequently, “slow to get up” and “clutching of head” were poor predictors of diagnosed concussions. Bruce et al. (2018) speculated these signs, rather than reflecting concussive injury, may reflect an attempt to draw a penalty or lesser forces experienced at the head^20^. Although injury diagnosis in the current study was unknown, our finding that players with visible signs experienced greater head impact severity support the notion that any player exhibiting visible sign of concussion should be removed from play and receive appropriate medical attention.

We found no evidence to indicate that the severity of head impacts depended on the playing zone where the hit occurred. More head impacts occurred in the offensive zone, but there were no differences in head impact severity between playing zones. Previous studies have reporting conflicting evidence on whether the severity depends on playing zone. Swenson et al. (2022) reported that male youth athletes reached higher speeds in the neutral zone resulting in greater head linear accelerations and rotational velocities at impact^48^. However, Hutchison et al. (2015b) found that concussive impacts in male professional hockey most often occurred in the injured player’s defensive zone (45%), followed by the offensive (34%) and neutral (21%) zones^49^. Further investigation is required on whether hits tend to be more severe in specific playing zones at different levels of play.

We also found no evidence that head impact severity depended on the object striking the head. Collectively, over 80% of head impacts involved the head being struck by an opponent’s upper limb (44% of all cases) or the head striking the boards or glass (36% of cases). There were no differences in the severity of impacts to the head from being struck by an opponent’s “shoulder/upper arm,” “elbow/forearm,” or “hand.” Potvin et al. (2019) examined the severity and duration of linear and rotational head accelerations when players delivered padded shoulder-, elbow-, and hand-to-head impacts “as hard as they were comfortable in delivering” to an instrumented kickboxing dummy^50^. They found that mean peak linear and rotational head accelerations were up to 2.1-fold greater for the hand and 1.9-fold greater for the elbow than shoulder. Our current results suggest that, during real-life game play in men’s university hockey, impacts delivered by the shoulder, elbow, and hand create similar peak head accelerations and rotational velocities. Head-to-glass collisions were just as severe, and much more common, than head-to-board collisions. Tuominen et al. (2017) and Schmitt et al. (2018) showed that modifications to the rink may reduce impact severity and injury risk^51,52^. Our findings suggest that additional studies are required to evaluate the stiffness of the glass/boards and its effect on head accelerations. Previous studies which found differences in head impact severity either (1) reported small differences in mean magnitudes (< 2 g or < 200 rad/s^2^), where the clinical significance is unclear, or (2) examined factors at high impact magnitudes (e.g., > 20 g threshold or at the 95th percentile), excluding common low-magnitude impact events^11,13,22,24^.

We found no association between head impact severity and anticipation of the collision. Mihalik et al. (2010a) also reported no differences in mean head accelerations between anticipated and unanticipated head impacts in youth hockey (aged 14)^22^. Furthermore, Eliason et al. (2022) found that more experience in delivering and receiving body checks did not protect minor hockey players (aged 15–17) against injury, including concussion^53^. Future research is required to evaluate the protective value of anticipatory responses and player training in reducing the frequency and severity of head impacts and injury in hockey.

Our study has several strengths. While previous studies had examined male youth hockey, ours is the first to combine head kinematics from helmet sensors with video footage to identify the most common and severe head impact scenarios in men’s university ice hockey. We also extend previous research by examining how impact severity depended on the specific object that impacts the head, puck possession, and visible signs of concussion. We recorded game play with a five-camera system, whereas most studies have used only one camera, and we used the video footage to verify that every case we examined involved a direct impact to the head.

Our study also has important limitations. First, we only analyzed data from the home games of a single men’s university ice hockey team. Therefore, results from this study may not apply to other contexts (e.g., practices; women’s ice hockey; other teams, leagues, and levels of play). Second, we observed substantial to perfect inter-rater reliability (k_n_ > 0.60) for most questionnaire items used in our analysis. However, caution should be used when interpreting “looking in the direction of the collision,” as only fair agreement was achieved (TPA = 67%, k_n_ = 0.31). Third, we included only the portion of head impacts having verified matches between video and sensor data. However, we have no reason to believe that the head impacts analyzed in the current study are not representative of all head impacts in the games we studied. Fourth, we reported peak head kinematics as proxy measures for head impact severity and consequently the degree of brain trauma. Future research should consider estimating brain tissue strain, using finite element models, which has been shown to have the closest association with brain injury^8,25,54^. Fifth, we only included head impacts observed by six research assistants who watched the game from different angles around the rink, and it is likely some head impacts were missed by the observers. However, our approach ensured that we only included direct head impact events in our analysis. We did not review all sensor-recorded events using the video footage, since previous research has shown that sensor-recorded events often do not correspond to a direct head impact. For example, Wilcox et al. (2014b) used helmet-mounted sensors (HITS) and recorded 1965 impact events across 12 home games in a single season, yet only 270 head impacts were verified on video^24^. Finally, the accuracy of helmet-mounted sensors in reflecting head accelerations and velocities may be affected by factors such as helmet fit, sensor location, and vibration of the helmet shell^35,38–40^. To minimize these effects, we standardized the helmet model and sensor placement. Future studies should consider using instrumented mouthguard sensors, which are less error prone and are associated with improved skull coupling^17,34–36^.

## Data Availability

The datasets analyzed during the current study are available from the corresponding author upon reasonable request.

## References

[1] Cusimano MD (2013). Mechanisms of team-sport-related brain injuries in children 5 to 19 years old: Opportunities for prevention. PLoS ONE.

[2] Chandran A (2022). Epidemiology of concussions in National Collegiate Athletic Association (NCAA) sports: 2014/15–2018/19. Am. J. Sports Med..

[3] Zuckerman SL (2015). Epidemiology of sports-related concussion in NCAA athletes from 2009–2010 to 2013–2014: Incidence, recurrence, and mechanisms. Am. J. Sports Med..

[4] Agel J, Dompier TP, Dick R, Marshall SW (2007). Descriptive epidemiology of collegiate men’s ice hockey injuries: National Collegiate Athletic Association injury surveillance system, 1988–1989 through 2003–2004. J. Athl. Train..

[5] Agel J, Harvey EJ (2010). A 7-year review of men’s and women’s ice hockey injuries in the NCAA. Can. J. Surg..

[6] Delaney JS, Al-Kashmiri A, Correa JA (2014). Mechanisms of injury for concussions in university football, ice hockey, and soccer. Clin. J. Sport Med..

[7] Hutchison MG, Comper P, Meeuwisse WH, Echemendia RJ (2015). A systematic video analysis of National Hockey League (NHL) concussions, part II: How concussions occur in the NHL. Br. J. Sports Med..

[8] Karton, C. & Hoshizaki, T. B. Concussive and subconcussive brain trauma: the complexity of impact biomechanics and injury risk in contact sport. In *Handbook of Clinical Neurology* Vol. 158 (eds Hainline, B. & Stern, R. A.) 39–49. 10.1016/B978-0-444-63954-7.00005-7 (Elsevier, 2018). 10.1016/B978-0-444-63954-7.00005-7 30482368

[9] Montenigro PH (2017). Cumulative head impact exposure predicts later-life depression, apathy, executive dysfunction, and cognitive impairment in former high school and college football players. J. Neurotrauma.

[10] Fickling SD (2021). Subconcussive brain vital signs changes predict head-impact exposure in ice hockey players. Brain Commun..

[11] Brainard LL (2012). Gender differences in head impacts sustained by collegiate ice hockey players. Med. Sci. Sports Exerc..

[12] Mihalik JP, Guskiewicz KM, Jeffries JA, Greenwald RM, Marshall SW (2008). Characteristics of head impacts sustained by youth ice hockey players. Proc. Inst. Mech. Eng. P.

[13] Mihalik JP (2012). Head impact biomechanics in youth hockey: Comparisons across playing position, event types, and impact locations. Ann. Biomed. Eng..

[14] Mihalik JP, Wasserman EB, Teel EF, Marshall SW (2020). Head impact biomechanics differ between girls and boys youth ice hockey players. Ann. Biomed. Eng..

[15] Wilcox BJ (2014). Head impact exposure in male and female collegiate ice hockey players. J. Biomech..

[16] Post A, Blaine Hoshizaki T (2015). Rotational acceleration, brain tissue strain, and the relationship to concussion. J. Biomech. Eng..

[17] Tierney G (2021). Concussion biomechanics, head acceleration exposure and brain injury criteria in sport: A review. Sports Biomech..

[18] Cortes N (2017). Video analysis verification of head impact events measured by wearable sensors. Am. J. Sports Med..

[19] Wu LC (2018). Detection of American football head impacts using biomechanical features and support vector machine classification. Sci. Rep..

[20] Bruce, J . M. *et al.* Development of a risk prediction model among professional hockey players with visible signs of concussion. *Br. J. Sports Med.* **52**(17), 1143–1148. 10.1136/bjsports-2016-097091(2018). 10.1136/bjsports-2016-097091 28377444

[21] Swenson AG, Pritchard NS, Miller LE, Urban JE, Stitzel JD (2023). Characterization of head impact exposure in boys’ youth ice hockey. Res. Sports Med..

[22] Mihalik JP (2010). Collision type and player anticipation affect head impact severity among youth ice hockey players. Pediatrics.

[23] Mihalik JP (2010). Effect of infraction type on head impact severity in youth ice hockey. Med. Sci. Sports Exerc..

[24] Wilcox BJ (2014). Head-impact mechanisms in men’s and women’s collegiate ice hockey. J. Athl. Train.

[25] Post A (2021). Comparison of frequency and magnitude of head impacts experienced by Peewee boys and girls in games of youth ice hockey. Comput. Methods Biomech. Biomed. Eng..

[26] Butterfield J (2023). A video analysis examination of the frequency and type of head impacts for player positions in youth ice hockey and FE estimation of their impact severity. Sports Biomech..

[27] Goulet C (2016). The incidence and types of physical contact associated with body checking regulation experience in 13–14 year old ice hockey players. IJERPH.

[28] Malenfant S, Goulet C, Nadeau L, Hamel D, Emery CA (2012). The incidence of behaviours associated with body checking among youth ice hockey players. J. Sci. Med. Sport.

[29] Williamson RA (2022). Does increasing the severity of penalties assessed in association with the “zero tolerance for head contact” policy translate to a reduction in head impact rates in youth ice hockey?. Clin. J. Sport Med..

[30] Aguiar Olivia M. G. (2020). American society of biomechanics journal of biomechanics award 2019: Circumstances of head impacts in men’s university ice hockey. Journal of Biomechanics.

[31] Kindschi K, Higgins M, Hillman A, Penczek G, Lincoln A (2017). Video analysis of high-magnitude head impacts in men’s collegiate lacrosse. BMJ Open Sport Exerc. Med..

[32] Muise DP, MacKenzie SJ, Sutherland TM (2016). Frequency and magnitude of head accelerations in a Canadian interuniversity sport football team’s training camp and season. Int. J. Athl. Ther. Train..

[33] King, D., Hume, P., Gissane, C., Brughelli, M. & Clark, T. The influence of head impact threshold for reporting data in contact and collision sports: Systematic review and original data analysis. *Sports Med.* **46**, 151–169. 10.1007/s40279-015-0423-7 (2016). 10.1007/s40279-015-0423-7 26545363

[34] Cummiskey B (2017). Reliability and accuracy of helmet-mounted and head-mounted devices used to measure head accelerations. Proc. Inst. Mech. Eng. P.

[35] Jadischke R, Viano DC, Dau N, King AI, McCarthy J (2013). On the accuracy of the head impact telemetry (HIT) System used in football helmets. J. Biomech..

[36] Wu LC (2016). In vivo evaluation of wearable head impact sensors. Ann. Biomed. Eng..

[37] Patton DA (2016). A review of instrumented equipment to investigate head impacts in sport. Appl. Bionics Biomech..

[38] Allison MA, Kang YS, Maltese MR, Bolte JH, Arbogast KB (2015). Measurement of Hybrid III head impact kinematics using an accelerometer and gyroscope system in ice hockey helmets. Ann. Biomed. Eng..

[39] Knowles BMK, Yu H, Dennison CR (2017). Accuracy of a wearable sensor for measures of head kinematics and calculation of brain tissue strain. J. Appl. Biomech..

[40] Campbell KR (2016). Laboratory evaluation of the gForce Tracker™, a head impact kinematic measuring device for use in football helmets. Ann. Biomed. Eng..

[41] Allison MA, Kang YS, Bolte JHI, Maltese MR, Arbogast KB (2014). Validation of a helmet-based system to measure head impact biomechanics in ice hockey. Med. Sci. Sports Exerc..

[42] Weaver AA, Danelson KA, Stitzel JD (2012). Modeling brain injury response for rotational velocities of varying directions and magnitudes. Ann. Biomed. Eng..

[43] Aguiar, O.M.G. *et al*. Head Impact Video Evaluation (HIVE) Questionnaire. Zenodo. 10.5281/zenodo.7847675 (2023).

[44] Brennan, R. L. & Prediger, D. J. Coefficient kappa: some uses, misuses, and alternatives. *Educ. Psychol. Meas.* **41**, 687–699. (1981).

[45] Echemendia, R. J. *et al*. Can visible signs predict concussion diagnosis in the National Hockey League? *Br. J. Sports Med.* **52**(17), 1149–1154. 10.1136/bjsports-2016-097090 (2018). 10.1136/bjsports-2016-097090 28377445

[46] Dong, M. Generalized linear mixed model add-in. JMP User Community https://community.jmp.com/t5/JMP-Add-Ins/Generalized-Linear-Mixed-Model-Add-in/ta-p/284627 (2020).

[47] Hockey Canada playing rules 2022-2024. https://cdn.hockeycanada.ca/hockey-canada/Hockey-Programs/Officiating/Downloads/rulebook_casebook_e.pdf (2022).

[48] Swenson, A. G. *et al*. Head Kinematics in Youth Ice Hockey by Player Speed and Impact Direction, *J. Appl. Biomech.* **38**(4), 201–209. 10.1123/jab.2021-0331(2022). 10.1123/jab.2021-0331 35894976

[49] Hutchison, M. G., Comper, P., Meeuwisse, W. H. & Echemendia, R. J. A systematic video analysis of National Hockey League (NHL) concussions part I: who when where and what? *BJSM*. **49**(8), 547–551 10.1136/bjsports-2013-092234 (2015b). 10.1136/bjsports-2013-092234 23766438

[50] Potvin Brigitte M. (2019). A comparison of the magnitude and duration of linear and rotational head accelerations generated during hand-, elbow- and shoulder-to-head checks delivered by hockey players. Journal of Biomechanics.

[51] Tuominen, M. *et al.* Concussion in the international ice hockey World Championships and Olympic Winter Games between 2006 and 2015, British *J. of Sports Med.* **51**(4), 244–252. 10.1136/bjsports-2016-097119 (2017). 10.1136/bjsports-2016-097119 28148512

[52] Schmitt, K.-U., Muser, M. H., Thueler, H. & Bruegger, O. Crash-test dummy and pendulum impact tests of ice hockey boards: greater displacement does not reduce impact, *Br. J. Sports Med.* **52**(1), 41–46. 10.1136/bjsports-2017-097735 (2018). 10.1136/bjsports-2017-097735 PMC5754856 29084724

[53] Eliason, P. H. *et al.* Bodychecking experience and rates of injury among ice hockey players aged 15–17 years, *Can. Med. Assoc. J.* **194**(24) E834–E842. 10.1503/cmaj.211718(2022) 10.1503/cmaj.211718 PMC9261946 35725006

[54] Zhang L, Yang KH, King AI (2004). A proposed injury threshold for mild traumatic brain injury. J. Biomech. Eng..

